# Plasma glycoproteomic biomarkers identify metastatic melanoma patients with reduced clinical benefit from immune checkpoint inhibitor therapy

**DOI:** 10.3389/fimmu.2023.1187332

**Published:** 2023-06-14

**Authors:** Chad Pickering, Paul Aiyetan, Gege Xu, Alan Mitchell, Rachel Rice, Yana G. Najjar, Joseph Markowitz, Lisa M. Ebert, Michael P. Brown, Gonzalo Tapia-Rico, Dennie Frederick, Xin Cong, Daniel Serie, Klaus Lindpaintner, Flavio Schwarz, Genevieve M. Boland

**Affiliations:** ^1^ InterVenn Biosciences, South San Francisco, CA, United States; ^2^ Department of Medicine, University of Pittsburgh Medical Center (UPMC) Hillman Cancer Center, Pittsburgh, PA, United States; ^3^ Department of Cutaneous Oncology, H. Lee Moffitt Cancer Center and Research Institute, Tampa, FL, United States; ^4^ Immuno-Oncology Program, H. Lee Moffitt Cancer Center and Research Institute, Tampa, FL, United States; ^5^ Centre for Cancer Biology, South Australia (SA) Pathology and University of South Australia, Adelaide, SA, Australia; ^6^ Cancer Clinical Trials Unit, Royal Adelaide Hospital, Adelaide, SA, Australia; ^7^ Adelaide Medical School, The University of Adelaide, Adelaide, SA, Australia; ^8^ Department of Surgery, Massachusetts General Hospital, Boston, MA, United States

**Keywords:** glycosylation, immune checkpoint inhibitors, biomarker, liquid biopsy, glycoproteomics, melanoma

## Abstract

The clinical success of immune-checkpoint inhibitors (ICI) in both resected and metastatic melanoma has confirmed the validity of therapeutic strategies that boost the immune system to counteract cancer. However, half of patients with metastatic disease treated with even the most aggressive regimen do not derive durable clinical benefit. Thus, there is a critical need for predictive biomarkers that can identify individuals who are unlikely to benefit with high accuracy so that these patients may be spared the toxicity of treatment without the likely benefit of response. Ideally, such an assay would have a fast turnaround time and minimal invasiveness. Here, we utilize a novel platform that combines mass spectrometry with an artificial intelligence-based data processing engine to interrogate the blood glycoproteome in melanoma patients before receiving ICI therapy. We identify 143 biomarkers that demonstrate a difference in expression between the patients who died within six months of starting ICI treatment and those who remained progression-free for three years. We then develop a glycoproteomic classifier that predicts benefit of immunotherapy (HR=2.7; p=0.026) and achieves a significant separation of patients in an independent cohort (HR=5.6; p=0.027). To understand how circulating glycoproteins may affect efficacy of treatment, we analyze the differences in glycosylation structure and discover a fucosylation signature in patients with shorter overall survival (OS). We then develop a fucosylation-based model that effectively stratifies patients (HR=3.5; p=0.0066). Together, our data demonstrate the utility of plasma glycoproteomics for biomarker discovery and prediction of ICI benefit in patients with metastatic melanoma and suggest that protein fucosylation may be a determinant of anti-tumor immunity.

## Introduction

Over the last decade, ICI therapeutics have significantly advanced the clinical management and outcome of patients with a range of malignancies, including metastatic melanoma (1, 2). However, only 30-40% of patients obtain sustained clinical benefit from single agent ICI therapy such as pembrolizumab or nivolumab (3, 4). While combination treatment of nivolumab with ipilimumab has resulted in higher response rates of up to 58% with unprecedented durability (5), it is also associated with an incidence of 59% of grade 3-to-4 immune-related adverse events (6). Therefore, considerable efforts have been made to discover predictive biomarkers for the early identification of patients who are unlikely to benefit from ICI treatment or may experience severe adverse events, and to offer alternative therapies to these patients in a timely manner (7, 8). Assessment of PD-L1 expression in tumor tissue and quantification of tumor mutational burden have found limited utility as indicators of durable clinical benefit in metastatic melanoma and are not approved by the FDA as companion diagnostics (9–13). Likewise, a number of other genomic, transcriptomic and multiomic approaches have been investigated as predictors of response to ICI therapies (14–18). In addition, gene expression signatures including immune-predictive score (IMPRES) and IFN-γ-response genes (TiME) have been proposed as predictors of ICI response in metastatic melanoma (19, 20). While these approaches are promising, they have not yet demonstrated broad applicability as there is debate around their consistency and reproducibility across cohorts (21–24).

The clinical utility of molecular tumor profiling is also limited by the intrinsic heterogeneity of tumor samples (25) and the availability of adequate tissue obtained through invasive procedures. Liquid biopsies have emerged as a desirable alternative for patient stratification as they are minimally invasive, convenient for serial sampling, and can be informative of the functional state of immune cells exposed to ICI therapy (26–29). Whereas peripheral immune cells and plasma factors contribute to antitumor responses modulated by ICI treatment, previous studies focus on only small groups of proteins (30–33). Moreover, the impact of post-translational modifications of circulating proteins on ICI efficacy has not been evaluated in a systematic and scalable way to date.

Here, we interrogate a set of plasma glycoproteins in metastatic melanoma patients before receiving ICI treatment using a novel platform that employs artificial intelligence to analyze data generated by targeted mass spectrometry. We identify glycopeptide markers that show differential expression in patients with short OS as compared to those with favorable OS outcomes and build a classifier predicting the likelihood of benefit from ICI therapy. We also provide insights of the underlying molecular mechanisms by investigating specific patterns of glycosylation observed in the set of biomarkers associated with likelihood of ICI therapy benefit. Altogether our data may inform the development of diagnostic tests to guide treatment decisions.

## Materials and methods

### Sample collection and clinical data

A cohort of 202 patients with metastatic melanoma treated with first or second-line anti-PD-1 monotherapy or anti-PD-1/anti-CTLA-4 combination therapy (referred to as the “discovery cohort”) was recruited as part of studies conducted at the Massachusetts General Hospital (MGH). Pre-treatment samples (obtained from patients prior to receiving ICI therapy) were collected under MGH Institutional Review Board protocols 12-488 and 11-181. Written informed consent was obtained from each patient. Subject benefit from ICI therapy was assessed using OS. An additional cohort of 27 patients (referred to as the “independent validation cohort”) was recruited as part of studies conducted at Royal Adelaide Hospital in which patients with metastatic melanoma were treated with first or second-line anti-PD-1 monotherapy (pembrolizumab or nivolumab) or anti-PD-1/anti-CTLA-4 combination therapy (nivolumab/ipilimumab). Written informed consent was obtained from each patient, and the study was approved by the Central Adelaide Local Health Network Human Research Ethics Committee (protocol HREC/16/RAH/95). Plasma samples from both cohorts were stored at -80°C and thawed at the time of the glycoproteomic analysis. Available clinical data included age at diagnosis, sex, *BRAF* mutation status, metastatic stage, lactate dehydrogenase (LDH) levels, Eastern Cooperative Oncology Group (ECOG) performance-status, prescribed ICI regimen, date of death, and date of disease progression. A progression event was defined as at least a 20% increase of the sum of longest diameters of existing lesions compared to the minimum sum of longest diameters during treatment, or manifestation of new lesions, death, or last contact at which time the event is censored, whichever occurred first.

### Chemicals and reagents

Pooled human plasma (MilliporeSigma, Burlington, MA) was used for quality control, assay normalization, and calibration. Dithiothreitol (DTT) and iodoacetamide (IAA) were purchased from MilliporeSigma (St. Louis, MO). Mass spectrometry grade trypsin/Lys-C protease mix, formic acid, and acetonitrile were purchased from Thermo Fisher Scientific (Waltham, MA). Stable isotope-labeled peptide internal standards were purchased from Vivitide (Gardner, MA).

### Liquid chromatography-mass spectrometry analysis

Plasma samples were heat-denatured at 100°C for five minutes followed by reduction with DTT, alkylation with IAA, quenching with DTT, and digestion with trypsin/Lys-C protease mix in a water bath at 37°C for 18 hours. To quench the digestion, formic acid was added to each sample to a final concentration of 1% (v/v). Digested plasma samples were spiked with stable isotope-labeled peptide internal standards before being injected into a 6495C triple quadrupole mass spectrometer (Agilent, Santa Clara, CA) equipped with a 1290 Infinity ultra-high-pressure liquid chromatography system (Agilent) mounting a Peptide HSS T3 column (2.1 mm internal diameter x 150 mm length, 1.8 µm particle size) (Waters, Milford, MA). Separation of peptides and glycopeptides was performed using a 49-minute binary gradient. The aqueous mobile phase A was 0.1% formic acid in water (v/v), and the organic mobile phase B was 0.1% formic acid in acetonitrile (v/v). The flow rate was set at 0.5 mL/min. Electrospray ionization was used as the ionization source and was operated in positive ion mode. The triple quadrupole MS was operated in dynamic multiple reaction monitoring (dMRM) mode, with modifications and improvements from a previous method (34). Samples were injected in a randomized fashion balanced on clinical attributes, and reference pooled plasma samples were injected interspersed with test samples to allow for correction of within-run drift of baseline signal.

### Glycoproteomic data analysis

PB-NET, a peak integration software developed in-house, was used to integrate peaks and obtain raw abundance for peptides and glycopeptides (35). Raw abundance of peptide markers was normalized by using spiked-in heavy isotope-labeled internal peptide standards to determine peptide concentration. Relative abundance was determined for glycopeptides with one or two types of glycans at a site by calculating the quotient of the raw abundance of the glycopeptide and the raw abundance of a selected non-glycosylated peptide from the same protein. Site occupancy was determined for glycopeptides with the same peptide sequence and more than two types of glycans identified at a given glycosylation site, by calculating the fractional abundance of any glycan and the aggregate abundance of all glycan types observed at that site. For each glycopeptide biomarker, the product of its site occupancy or relative abundance and the corresponding peptide concentration was used to calculate approximate glycopeptide concentration, also referred to as normalized abundance. Concentration was determined for 521 glycopeptides, 443 of which are based on site occupancy and 78 on relative abundance, and for 75 peptides, totaling 596 unique concentration-normalized biomarkers. Relative abundance was determined for 532 unique glycopeptides. Univariate age- and sex-adjusted Cox regression with respect to OS was performed using both relative abundance-normalized features and concentration-normalized features to identify statistically significant association of glycopeptides and non-glycosylated peptides with OS, and correction for multiple testing was performed using the false discovery rate (FDR) via the Benjamini and Hochberg method (36). To develop classifiers, five-fold repeated cross-validated LASSO-regularized Cox regression was performed. Resulting risk scores for each patient were dichotomized into “likely to benefit from ICI therapy” and “unlikely to benefit from ICI therapy” groups by a threshold chosen where the concordance index is maximized in the discovery cohort’s training set. The same threshold was used in the discovery cohort’s validation and test set, as well as in the independent validation cohort. The proportional hazards assumption was met for all applications of Cox regression performed in the analysis. All analyses were conducted in R version 4.2.2 (Vienna, Austria) (37).

### Glycopeptide marker interpretation

Differentially expressed glycopeptides in relative abundance with respect to OS at p<0.05 were analyzed by type of glycosylation. Asparagine (N)-linked glycans and O-linked glycopeptides were then stratified based on the number of fucose or sialic acid units. GraphPad Prism (GraphPad Software, Boston, MA) was used for data visualization and statistical analysis. A site-specific monomer weight feature for N-glycopeptides was calculated across the entire panel of markers by determining the average number of any one specific monosaccharide at a given glycosylation site, weighted by glycan species site occupancy. Five-fold repeated cross-validated LASSO-regularized Cox regression based only on fucose-dependent monomer weight features that achieved FDR<0.05 in univariate age- and sex-adjusted Cox regression was performed, and a risk score threshold was chosen using the same method as described above. The same training, validation, and test sets of the discovery cohort were used as in the other cross-validated Cox model described above.

### Functional pathway analysis

Peptides associated with OS in differential expression analysis were mapped to their cognate Entrez gene identifiers. Pathway enrichment analysis (38) was performed using the enrichPathway routine defined in the ReactomePA package (39, 40). Based on functionally annotated reaction pathways in the Reactome Knowledgebase, the probability of an erroneous prediction using the hypergeometric test was estimated by the enrichPathway function (41). Correction for multiple testing was applied using the Benjamini and Hochberg method (36). Hierarchical clustering of enriched terms that relies on their pairwise similarities was performed to explore similarity between functional modules using Jaccard’s similarity index and the average agglomeration method, and then implemented in the treeplot function of the enrichplot R package. Disease ontology (DO) enrichment analysis was also performed to investigate possible relationships of biomarkers of interest to biomarkers known to be associated with neoplastic disease processes (42). Enrichment against the curated Network of Cancer-causing Genes was performed by analyzing semantic similarities among DO terms and by hypergeometric tests to find the probability of erroneously finding a disease entity to be functionally enriched (43). All analyses were performed using R version 4.2.2. Results were plotted using functions defined in the enrichplot R package (44).

## Results

### Description of the cohorts

**Figure 1:**
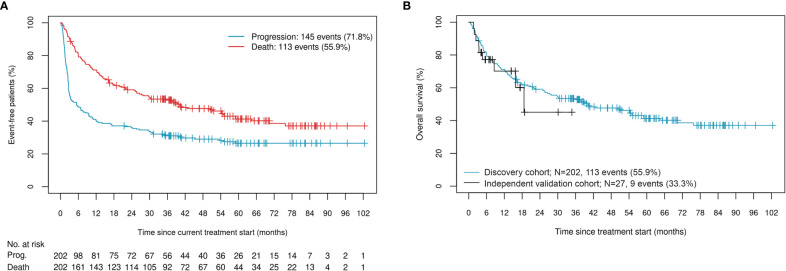
**(A)** Occurrence of events in the discovery cohort; **(B)** OS and censoring distributions in the discovery and independent validation cohorts.

We assessed benefit of ICI therapy using OS. The discovery cohort of 202 patients was composed of 69% males with a median age of 65 years (IQR: 57, 73). Seventy-three percent of patients underwent first-line therapy, with 56% of all patients having a recorded death. Median time to progression for this cohort was 5.5 months (95% CI: 3.0, 9.9), while median time to death was over three years, at 40.1 months (95% CI: 27.3, 59.0) (**Figure 1A**). Sixty-three percent of patients had cutaneous melanoma, 17% had melanoma of unknown primary, and the remainder were characterized as either mucosal, uveal, or acral subtype. At the start of treatment, 73% of the cohort was staged as either M1c or M1d, 33% of patients carried *BRAF* mutations, 44% had an LDH (units/L) value that exceeded the upper limit of normal (ULN), and 40% had an ECOG performance status above 0 (**Table 1**). As expected, patients with non-cutaneous melanoma, higher LDH, and higher ECOG performance status exhibited shorter median time to death, while those carrying *BRAF* mutations exhibited a longer median time to death (**Supplementary Figure 1**). The sample set was randomly divided and stratified by immunotherapy regimen, melanoma subtype, and early failure (death within six months of treatment start) or sustained control (progression-free for three years or more since initiation of ICI treatment) status. Forty percent of the cohort was used as a training set, 30% was used as a validation set on which to tune model hyperparameters, and the remaining 30% was used as a testing set. Allocation of samples to the three sets was found to be well-balanced across all demographic and clinical covariables listed in **Table 1** (confirmed by Chi-squared, Fisher’s Exact, Student t, or Wilcoxon rank-based tests, as appropriate).

**Table 1:** Demographic and clinical covariates in the discovery cohort.

Variable	Full cohort	Training set	Validation set	Test set
**Sample size**	202	79 (39)	59 (29)	64 (32)
**Male sex**	139 (69)	52 (66)	43 (73)	44 (69)
**Age, yrs.** (continuous)	65 (57, 73)	67 (57, 72)	64 (56, 74.5)	63.5 (57, 72.2)
Current ICI treatment				
Pembrolizumab monotherapy	109 (54)	43 (54)	32 (54)	34 (53)
Nivolumab with/without ipilimumab combination	93 (46)	36 (46)	27 (46)	30 (47)
Survival-related events				
Progression (PFS) event	145 (72)	57 (72)	40 (68)	48 (75)
Death (OS) event	113 (56)	42 (53)	32 (54)	39 (61)
Time to event, mos. (continuous)				
Progression	5.5 (3.0, 9.9)	6.9 (3.3, 23.9)	8.4 (2.7, 30.4)	3.2 (2.5, 9.3)
Death	40.1 (27.3, 59.0)	50.4 (30.3, NR)	36.1 (14.3, NR)	28.8 (16.2, NR)
*BRAF* status				
Positive/mutant	67 (33)	22 (28)	18 (31)	27 (42)
V600	57 (28)	20 (25)	16 (27)	21 (33)
Non-V600	9 (4)	2 (3)	2 (3)	5 (8)
Non-specific mutant	1 (0)	0 (0)	0 (0)	1 (2)
Negative/wild type	121 (60)	49 (62)	35 (59)	37 (58)
Missing	14 (7)	8 (10)	6 (10)	0 (0)
LDH (categorical)				
<ULN	105 (52)	36 (46)	29 (49)	40 (62)
1-2xULN	63 (31)	28 (35)	22 (37)	13 (20)
>2xULN	27 (13)	12 (15)	6 (10)	9 (14)
Missing	7 (3)	3 (4)	2 (3)	2 (3)
**LDH, units/L** (continuous)	206 (167, 281)	218 (168.8, 284.8)	206 (167, 274)	191.5 (164.8, 276.8)
M Stage				
M0	16 (8)	7 (9)	5 (8)	4 (6)
M1	186 (92)	72 (91)	54 (92)	60 (94)
M1a	9 (4)	3 (4)	2 (3)	4 (6)
M1b	31 (15)	17 (22)	5 (8)	9 (14)
M1c	84 (42)	28 (35)	30 (51)	26 (41)
M1d	62 (31)	24 (30)	17 (29)	21 (33)
ECOG performance status				
0	119 (59)	49 (62)	34 (58)	36 (56)
1	67 (33)	24 (30)	19 (32)	24 (38)
≥2	14 (7)	5 (6)	6 (10)	3 (5)
Missing	2 (1)	1 (1)	0 (0)	1 (2)
Melanoma subtype				
Cutaneous	127 (63)	51 (65)	37 (63)	39 (61)
Mucosal	20 (10)	8 (10)	5 (8)	7 (11)
Uveal	15 (7)	6 (8)	5 (8)	4 (6)
Acral	5 (2)	1 (1)	1 (2)	3 (5)
Unknown primary	35 (17)	13 (16)	11 (19)	11 (17)
Line of therapy				
First-line	147 (73)	61 (77)	44 (75)	42 (66)
Second-line or later	54 (27)	17 (22)	15 (25)	22 (34)
Missing	1 (0)	1 (1)	0 (0)	0 (0)

Counts are followed by the appropriate column-wise percentage, while continuous variables are summarized by medians and either IQRs or, for time to event variables, 95% confidence limits (NR, not reached).

For additional validation of the classifier, we used an independent cohort composed of 70% males with a median age of 71 years (IQR: 66, 81), 85% of whom underwent pembrolizumab monotherapy (**Table 2**). Median time to death was 18.6 months (95% CI: 15.8, NR). While this cohort has less follow-up events than the discovery cohort, the distributions are comparable (**Figure 1B**).

**Table 2:** Demographic and clinical covariates in the independent validation cohort.

Variable	Full cohort
**Sample size**	27
**Male sex**	19 (70)
**Age, yrs.** (continuous)	71 (66, 81)
Current ICI treatment	
Pembrolizumab monotherapy	23 (85)
Ipilimumab/nivolumab combination	4 (15)
Survival-related events	
Progression (PFS) event	10 (37)
Death (OS) event	9 (33)
Time to event, mos. (continuous)	
Progression	NR (9.9, NR)
Death	18.6 (15.8, NR)
Best overall response	
Complete response	5 (19)
Partial response	5 (19)
Stable disease	6 (22)
Progressive disease	9 (33)
Missing	2 (7)

Counts are followed by the appropriate column-wise percentage, while continuous variables are summarized by medians and either IQRs or, for time to event variables, 95% confidence limits (NR, not reached).

### Identification of glycopeptide biomarkers associated with overall survival and early failure

We applied univariate age- and sex-adjusted Cox regression to identify peptides and glycopeptides associated with OS in the discovery cohort. We identified 64 concentration-normalized biomarkers that achieved FDR<0.05, 49 of which were glycopeptides.

We then assigned the label “early failure” (EF) to patients who died within six months of starting ICI treatment (n=40), “sustained control” (SC) to patients who neither progressed nor died in the first three years after ICI treatment start (n=56), and “other” to all patients that did not fit into these two categories (n=106) (**Supplementary Figures 2A, B**). We found 143 differentially expressed concentration-normalized biomarkers at FDR<0.05, demonstrating a stark difference in the glycoproteome of patients in the EF and SC groups (**Supplementary Figure 2C**).

### Generation of a classifier that predicts ICI benefit

**Figure 2:**
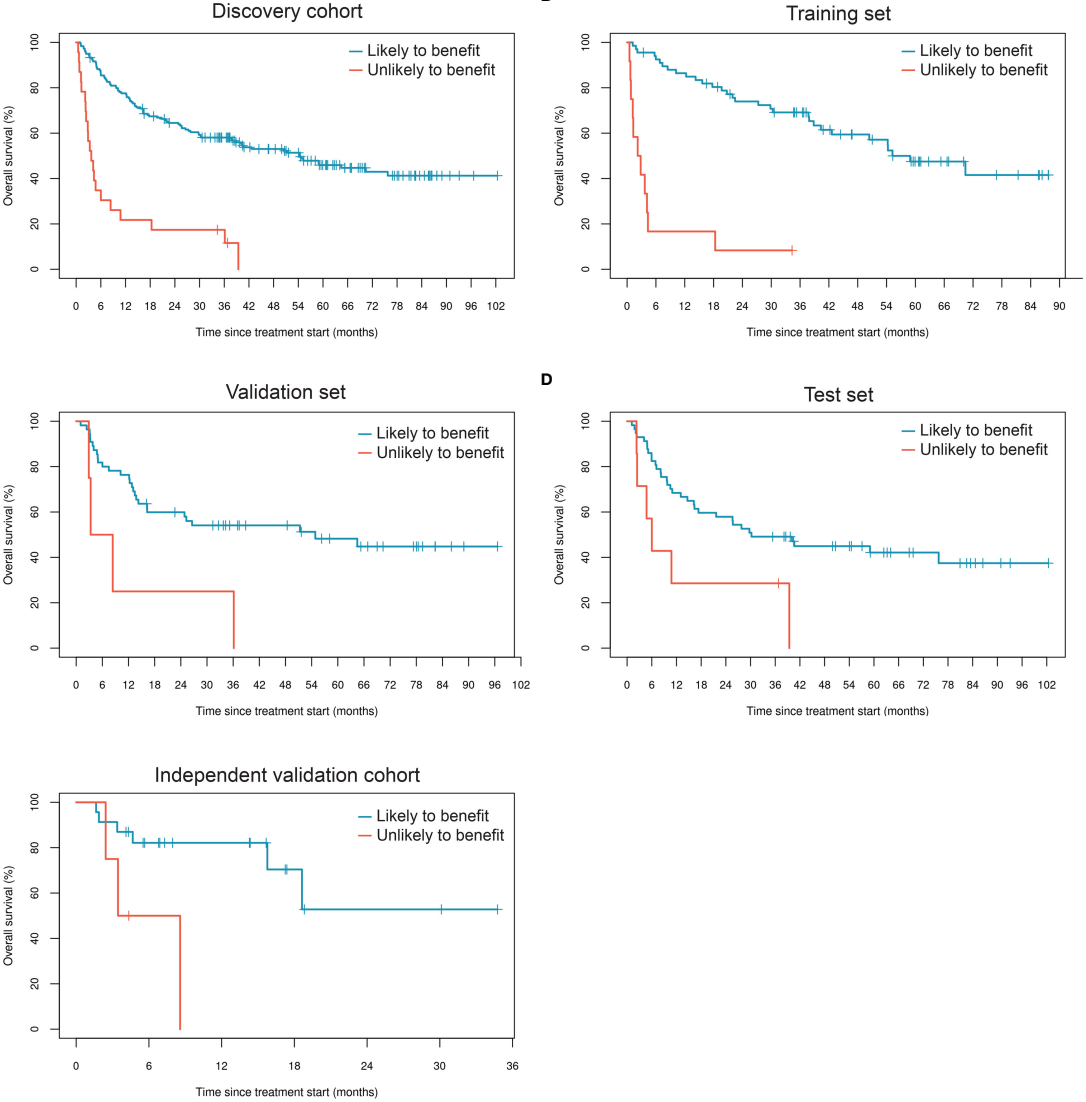
Performance of the glycoproteomic classifier in subsets of the discovery cohort **(A-D)** and in the independent validation cohort **(E)**.

We generated a LASSO-regularized Cox-based classifier for prediction of individuals with extended OS following ICI therapy. Out of all 596 concentration-normalized features, 14 were retained (13 glycopeptides and one peptide) and achieved an unadjusted HR=10.3 (p=4.5×10^-9^) in the training set, HR=3.9 (p=0.012) in the validation set, and HR=2.7 (p=0.026) in the test set (**Table 3**, **Figures 2A-D**). Patients classified as likely to benefit exhibited median OS of 54.3 months (95% CI: 37.9, NR), whereas patients classified as unlikely to benefit had median OS of 3.7 months (95% CI: 2.4, 10.8) in the full discovery cohort; in the test set, median OS reached 30.2 (95% CI: 16.4, NR) and 6.0 months (95% CI: 2.4, NR) for likely and unlikely to benefit groups, respectively. We then applied the classifier to the independent validation cohort. Remarkably, the model achieved comparable separability between the likely and unlikely to benefit classification groups relative to the discovery cohort (HR=5.6; p=0.027) (**Figure 2E**; **Table 3**). Although this cohort is small, these data serve as an independent validation of a melanoma-specific signature (**Table 3**).

**Table 3:** Performance of repeated five-fold cross-validated LASSO-regularized Cox regression-based classifier using 14 concentration-normalized biomarkers, stratified by cohort and subsets thereof.

Classifier prediction	Events/N	Median OS (95% CI)	HR (95% CI)	P-value
Discovery cohort (n=202)				
**Likely to benefit**	92/179	54.3 (37.9, NR)	Reference	
**Unlikely to benefit**	21/23	3.7 (2.4, 10.8)	5.1 (3.1, 8.4)	7.6×10^-11^
Discovery cohort: training set (n=79)				
**Likely to benefit**	31/67	55.2 (42.6, NR)	Reference	
**Unlikely to benefit**	11/12	2.5 (1.2, NR)	10.3 (4.7, 22.6)	4.5×10^-9^
Discovery cohort: validation set (n=59)				
**Likely to benefit**	28/55	54.8 (16.3, NR)	Reference	
**Unlikely to benefit**	4/4	5.8 (2.9, NR)	3.9 (1.4, 11.4)	0.012
Discovery cohort: test set (n=64)				
**Likely to benefit**	33/57	30.2 (16.4, NR)	Reference	
**Unlikely to benefit**	6/7	6.0 (2.4, NR)	2.7 (1.1, 6.6)	0.026
Discovery cohort: validation and test set (n=123)				
**Likely to benefit**	61/112	40.6 (24.8, NR)	Reference	
**Unlikely to benefit**	10/11	6.0 (3.3, NR)	3.2 (1.6, 6.3)	8.2×10^-4^
Independent validation cohort (n=27)				
**Likely to benefit**	6/23	NR (15.8, NR)	Reference	
**Unlikely to benefit**	3/4	6.0 (2.4, NR)	5.6 (1.2, 25.5)	0.027

NR, not reached.

Next, we estimated the statistical significance of all demographic and clinical variables with and without adjustment for glycoproteomic classifier prediction. This was ultimately done for variable selection and adjustment in a multivariate Cox regression analysis framework. Age, *BRAF* status, categorical LDH group, ECOG performance status, metastatic stage, melanoma subtype, and line of therapy all were retained in the model at p<0.15 in both analyses, with and without adjustment for classifier prediction (**Supplementary Table 1**). We then performed multivariate Cox regression analysis using the classifier prediction group as the primary independent variable and adjusting for the previously listed variables (**Table 4**). When applied to the combined validation and test sets in the discovery cohort, this model predicted that the risk of death for a patient who is classified as unlikely to benefit from ICI therapy based on their glycoproteomic risk score is about 2.1 times higher than for a patient classified as likely to benefit, adjusting for the demographic and clinical variables (95% CI: 0.9, 4.9). It is worth noting that age, categorical LDH group, ECOG performance status, melanoma subtype, and line of therapy were significant at p<0.05 in multivariate modeling without the inclusion of the classifier prediction variable. However, when the classifier prediction variable is included, the only predictors that remained statistically significant were classifier prediction (p=6.3×10^-5^), melanoma subtype (p=5.1×10^-4^), and line of therapy (p=5.9×10^-3^) (**Table 4**). Based on a partial likelihood ratio test to compare these two multivariate Cox models, there is sufficient evidence that the model that includes the classifier prediction provides a significantly better fit than the model without it (p=2.0×10^-4^). Moreover, while high LDH at the start of treatment was associated with increased risk of death, patients classified based on the glycoproteomic predictor as likely to benefit exhibited longer median OS compared to the group categorized as unlikely to benefit, regardless of the LDH category (**Supplementary Figure 3A**). The same pattern was observed with respect to *BRAF* mutation status: while carrying a *BRAF* mutation results in a modest association with lower risk of death, patients classified as likely to benefit are associated with longer median OS, regardless of *BRAF* mutation status (**Supplementary Figure 3C**).

**Table 4:** Performance of Cox regression-based classifier in the discovery cohort with multivariate adjustments.

	Full cohort (n=202)		Training set (n=79)		Validation + test sets (n=123)	
Variable	HR (95% CI)	P-value	HR (95% CI)	P-value	HR (95% CI)	P-value
**Classifier prediction (unlikely to benefit)**	3.355 (1.854, 6.069)	6.3×10^-5^	5.453 (1.704, 17.449)	4.3×10^-3^	2.128 (0.931, 4.861)	0.073
**Age (continuous years)**	1.017 (0.998, 1.035)	0.081	1.031 (0.998, 1.065)	0.066	1.007 (0.983, 1.031)	0.567
**Positive *BRAF* status (ref: negative)**	0.836 (0.522, 1.338)	0.455	0.843 (0.348, 2.041)	0.704	0.79 (0.434, 1.436)	0.439
**LDH category**		0.328		0.009		0.396
<ULN	Reference					
1-2xULN	1.073 (0.682, 1.688)	0.762	0.955 (0.405, 2.254)	0.916	0.994 (0.555, 1.779)	0.983
>2xULN	1.715 (0.934, 3.15)	0.082	4.151 (1.143, 15.073)	0.031	1.604 (0.739, 3.479)	0.232
**ECOG performance status**		0.222		0.236		0.007
0	Reference					
1	1.375 (0.894, 2.115)	0.147	1.564 (0.751, 3.256)	0.232	1.478 (0.839, 2.605)	0.176
≥2	1.941 (0.914, 4.121)	0.084	0.374 (0.062, 2.245)	0.282	5.511 (2.314, 13.127)	1.2×10^-4^
**M1 stage (ref: M0)**	2.146 (0.843, 5.464)	0.109	1.744 (0.439, 6.928)	0.429	4.932 (1.138, 21.375)	0.033
**Non-cutaneous subtype (ref: cutaneous)**	2.00 (1.353, 2.957)	5.1×10^-4^	3.026 (1.44, 6.358)	3.5×10^-3^	1.528 (0.898, 2.599)	0.118
**Not first-line therapy (ref: first-line)**	1.841 (1.192, 2.842)	5.9×10^-3^	1.981 (0.853, 4.603)	0.112	2.073 (1.212, 3.548)	7.8×10^-3^

Small numbers in certain variable subgroups justifies combining the validation and test sets for the purposes of this analysis.

### Fucosylation of N-glycopeptide markers is associated with reduced clinical benefit of ICI therapy

**Figure 3:**
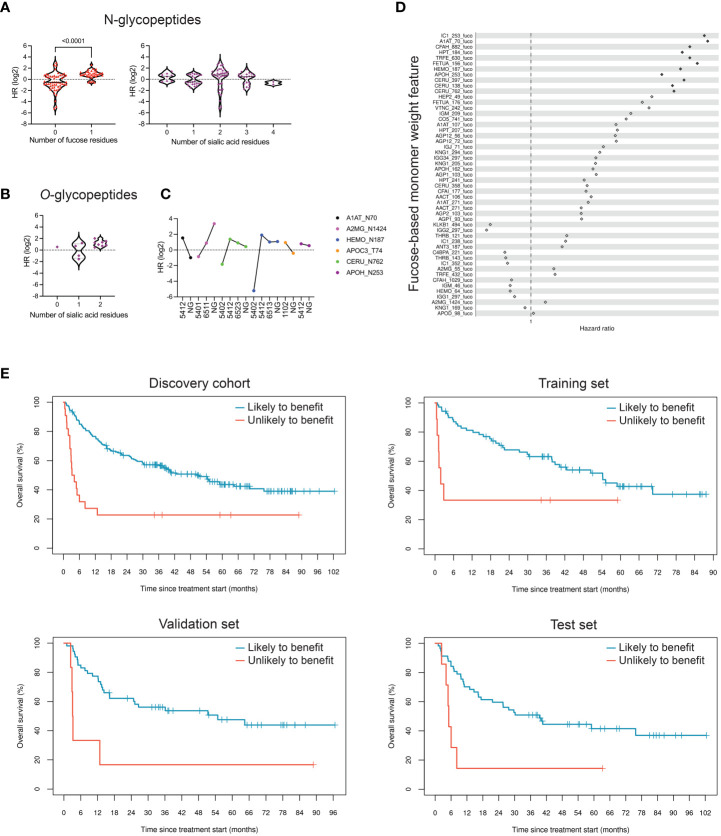
Fucosylation signatures in peripheral blood N-glycoproteins are associated with reduced clinical benefit of ICI therapy. **(A)** Differentially expressed glycopeptides (p<0.05), based on relative abundance measurements, in patients who are likely to benefit compared to those unlikely to benefit were classified based on their glycan structure. N-linked glycopeptides are separated into two groups based on the presence or absence of fucose; fucosylation is strongly associated with OS (p<0.0001). The number of sialic acid residues, however, is not associated with OS. HR, hazard ratio. **(B)** Di-sialylated *O*-glycopeptides are enriched in samples with reduced OS (p=0.14). **(C)** Effect of site occupancy on protein function in relation to likelihood to benefit. Lack of a glycan on site N70 of alpha-1-antitrypsin (A1AT_N70 NG) is associated with increased OS, whereas absence of glycosylation at the site N1424 of alpha-2-macroglobulin is associated with reduced OS. The four-digit number describes glycan composition (number of hexoses, *N*-acetyl-hexosamines, fucoses, and sialic acids, respectively). **(D)** Hazard ratios of 51 fucose-dependent monomer weight features derived from N-glycopeptides, sorted by age- and sex-adjusted Cox regression FDR. Hazard ratios of features that achieved FDR<0.05 are represented as filled-in diamonds. HR>1 represents association with shorter OS. **(E)** Kaplan-Meier curves showing performance of repeated five-fold cross-validated LASSO-regularized Cox regression-based classifier using 11 fucose-dependent features derived from N-glycopeptides that achieved FDR<0.05 in age- and sex-adjusted Cox regression analysis.

Glycopeptide markers showing differences in relative abundance at p<0.05 with respect to OS were selected for structural analysis. Of these markers, 91 were N-glycopeptides, all carrying complex-type glycans. Strikingly, when looking at fucose content, these markers separated into two distributions that aligned with benefit from ICI treatment (**Figure 3A**), with fucosylated glycopeptides prevalent in patients with shorter OS. Next, we analyzed sialic acid content in the same markers, as alterations in sialic acid density in tumor cells have been extensively described in connection with inhibition of immune cell function (45). However, we found little to no correlation between the number of sialic acid units and benefit from ICI treatment (**Figure 3A**). Instead, we observed a modest inverse correlation between the number of sialic acid units in *O*-linked glycopeptides and benefit of treatment (**Figure 3B**). Access to non-glycosylated forms of the peptides allowed us to also evaluate the relevance of site occupancy. As N-glycans are involved in protein folding and can affect interactions with other proteins, changes in site occupancy may dramatically change protein function. Interestingly, alpha-1-antitrypsin and alpha-2-macroglobulin exhibited opposite associations with OS with respect to glycosylation (**Figure 3C**).

To test the hypothesis that fucosylated N-glycopeptides are associated with lower likelihood of benefit from ICI therapy, we calculated “site-specific monomer weight features” that represent the average number of specific monosaccharides at a given site, weighted by glycopeptide occupancy. Of 51 fucose-dependent features across our full research assay, 11 were strongly associated with OS based on univariate age- and sex-adjusted Cox regression analysis (**Figure 3D**). All 11 features were ultimately retained in a LASSO-regularized Cox regression model that yielded a hazard ratio of 2.9 (p=0.016) in the training set of the discovery cohort (**Figure 3E**, **Table 5**). When applied to the validation and test sets, the model resulted in a hazard ratios of 3.8 (p=6.7×10^-3^) and 3.5 (p=6.6×10^-3^), respectively. Altogether, these data support the notion that the fucosylation status of specific circulating glycoproteins is a critical parameter that stratifies patients with regard to likelihood of benefit from ICI therapy.

**Table 5:** Performance of monomer weight model in the discovery cohort using 11 fucose-dependent features derived from N-glycopeptides that achieved FDR<0.05 in age- and sex-adjusted Cox regression analysis.

Classifier prediction	Events/N	Median OS (95% CI)	HR (95% CI)	P-value
Full discovery cohort (n=202)				
**Likely to benefit**	96/180	50.4 (36.1, 75.7)	Reference	
**Unlikely to benefit**	17/22	3.7 (2.8, 12.8)	3.1 (1.9, 5.3)	1.6×10^-5^
Discovery: training set (n=79)				
**Likely to benefit**	36/70	54.2 (37.9, NR)	Reference	
**Unlikely to benefit**	6/9	1.8 (1.2, NR)	2.9 (1.2, 7.0)	0.016
Discovery: validation set (n=59)				
**Likely to benefit**	27/53	54.8 (24.8, NR)	Reference	
**Unlikely to benefit**	5/6	3.2 (2.9, NR)	3.8 (1.4, 10.0)	6.7×10^-3^
Discovery: test set (n=64)				
**Likely to benefit**	33/57	39.4 (17.3, NR)	Reference	
**Unlikely to benefit**	6/7	5.1 (4.1, NR)	3.5 (1.4, 8.5)	6.6×10^-3^

NR, not reached.

### Functional pathway analysis uncovers a potential role of neutrophil degranulation

**Figure 4:**
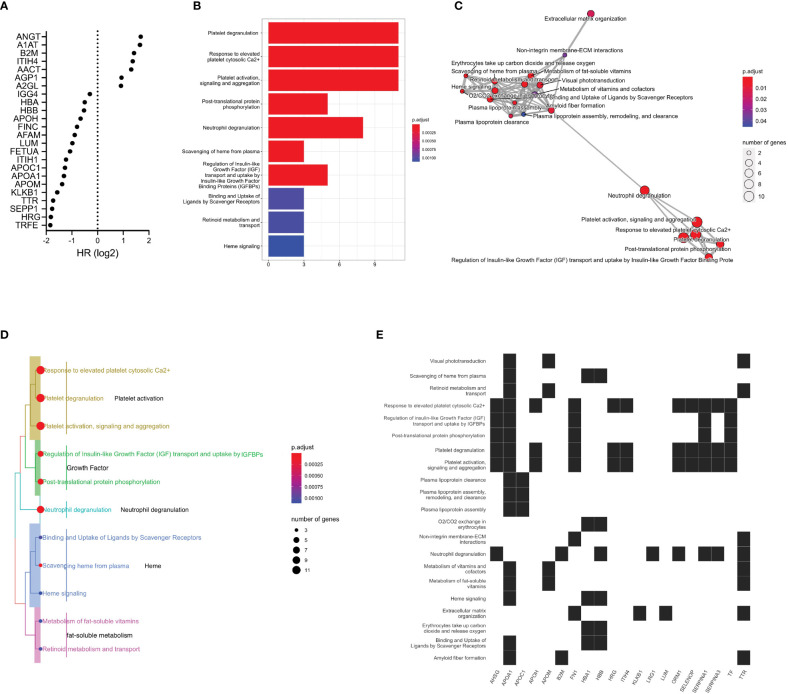
Functional pathway analysis of glycoproteins. **(A)** Differentially expressed plasma glycoproteins with respect to OS (p<0.05). **(B)** Significant functional pathways. The x-axis represents the number of genes in the respective pathways and the color indicates estimated p-values. **(C)** Enrichment map of significant functional pathways showing enriched terms organized into a network with edges connecting overlapping gene sets. Mutually overlapping gene sets, such as the platelet-related pathways, cluster together. The neutrophil degranulation pathway appears to bridge these pathways to transport- and metabolism-related pathways. **(D)** Enrichment treeplot was cut into five subtrees, each labeled with the most highly represented terms. The neutrophil degranulation pathway appears to be in close relationship to the growth-related and the platelet activation pathways. **(E)** Enrichment heatmap showing the links between genes and significant functional pathways.

We selected glycoproteins significantly associated with OS in concentration-normalized abundance for further analysis. Alpha-1-antitrypsin, alpha-1-antichymotrypsin, beta-2-microglobulin and leucine-rich alpha-2-glycoprotein 1 were found at higher concentration in patients with limited benefit from ICI treatment (**Figure 4A**). When applying pathway analysis to the corresponding genes, we identified mechanisms related to platelet activity and neutrophil function that included platelet degranulation, response to elevated platelet cytosolic Ca^2+^, and platelet activation, signaling, and aggregation (**Figure 4B** and **Supplementary Tables 4, 5**). The *APOA1* gene was found to be involved with the highest number of enriched pathways (16 functional pathways), followed by the *TTR* gene (8 significantly enriched pathways) (**Figures 4C, E**). With 11 genes in common, the platelet degranulation and response to elevated platelet cytosolic Ca^2+^ pathways appeared to cluster most closely (**Figure 4D**). Other closely related functional modules include the regulation of insulin-like growth factor and uptake and the post-translational protein phosphorylation pathways. To investigate any potential relationship between the significant feature genes and primary disease processes based on prior knowledge curation efforts, we performed disease ontology enrichment analysis, which revealed a weak relationship of the significant feature genes to melanoma (p=0.29).

## Discussion

Glycosylation is an abundant and complex form of post-translational modification of proteins that profoundly affects their structure, conformation, and function (46, 47). Recent advances in glycoproteomics have provided unique structural insights into the human glycoproteome and its regulation in health and disease (48–51). However, the application of glycosylated biomarkers in clinical settings has so far been limited by the technical challenges in generating and interpreting this information at scale. Our analytical platform detected plasma glycoproteomic markers that were differentially expressed in patients with metastatic melanoma treated with immunotherapy, with shorter or longer OS. These markers were used to generate classifiers that identified metastatic melanoma patients unlikely to derive clinical benefit from ICI therapy in two independent cohorts. We also discovered a specific fucosylation signature in plasma N-glycoproteins of patients that do not achieve a durable response to ICI therapy. Interestingly, higher levels of N-glycans containing fucose have been recently reported in association with reduced PFS in a glycomics study investigating responses to ICI (52). Elevated protein fucosylation, potentially resulting from increased expression of fucosyltransferases, has been described in the tumor microenvironment (TME) in melanoma (53). Increased core fucosylation promotes cell invasion and metastatic potential through the modulation of the stability of the L1CAM adhesin at the cell surface (54). Fucosylation also alters TGF-β signaling that drives immune-excluded TME phenotypes (55). Presence of fucose in the N-glycan of IgG1 significantly reduces its binding to FcγR and may be associated with lower efficacy of anti-tumor antibodies (56, 57). Moreover, fucosylation enhances display of PD-1 on the cell surface (58, 59). Fucosylation of the major histocompatibility complex-II HLA-DRB1 enhances CD4^+^ T cell immunity and enhances anti-PD-1 efficacy in a murine tumor model (60). We hypothesize that the same mechanisms that generate fucosylation signatures in circulating proteins detected in our assay may also lead to increased protein fucosylation in the TME that in turn affect responses to ICI treatment. Future research will address what factors drive changes in glycosylation in circulating proteins and how protein fucosylation modulates mechanisms that are relevant to ICI response in metastatic melanoma. We propose that glycopeptide markers may ultimately be used to distinguish immune-inflamed from immune-excluded or -desert TME phenotypes.

In addition to the fucosylation signature, we make other observations pertaining to mechanisms that may contribute to the efficacy of ICI treatment. Disialylated *O*-linked glycopeptides exhibited a modest association with shorter OS (**Figure 3B**); these glycan species likely correspond to disialylated core-1 *O*-glycan tetrasaccharides that bind to the immune-suppressive receptor Siglec-7 found on natural killer cells (61). A1AT glycosylation modulates binding to IL-8, a cytokine that stimulates neutrophil activation and is found to be elevated in patients with lower responses to ICI therapy (62–64). The leucine-rich alpha-2-glycoprotein 1 destabilizes tumor vessels and restricts immunotherapeutic potency (65). Beta-2-microglobulin imbalance may promote tumor escape from recognition by CD8+ T cells (57) and play a role in neutrophil degranulation (66). The neutrophil degranulation pathway likely facilitates a crosstalk between seemingly unrelated functional pathways (metabolism and transport-related pathways and the platelet activity related pathways).

When key clinical variables were stratified by the classifier prediction in the discovery cohort, a few patterns become clear. LDH, which partially defines prognosis in stage IV disease, is used as a surrogate marker of tumor burden (67). While having high LDH at the initiation of treatment is associated with increased risk of death, patients classified based on the glycoproteomic predictor as likely to benefit exhibited increased median OS compared to the group categorized as unlikely to benefit, regardless of the LDH category of a patient (**Supplementary Figure 3**). The same pattern is observed with respect to *BRAF* mutation status: while carrying a *BRAF* mutation shows a modest association with decreased risk of death, patients classified as likely to benefit are associated with longer median OS, regardless of *BRAF* mutation status. In contrast, whereas patients classified as unlikely to benefit had comparable median OS regardless of ECOG performance status, patients with ECOG performance status above 1 demonstrated short median OS regardless of glycoproteomic-based classification. These observations are additional evidence that the glycoproteomic classifier provides significant utility in identifying patients who are unlikely to benefit from ICI therapy, regardless of other physiological and clinical characteristics, and represents a substantial advance over current methods.

Whereas this is the largest study associating circulating glycoprotein profiles with benefit of ICI therapy in advanced melanoma to date, we recognize that our study has limitations. First, while the discovery cohort was limited to metastatic and unresectable melanoma, we accepted all such patients without further stratification as to the site of metastases, subtype, or functional status. Despite adjusting for these variables in multivariate analyses, the patient cohorts analyzed here may not perfectly represent the distributions of clinical and physiological characteristics found in larger populations. Secondly, while most patients received first-line single- or double-agent ICI therapy, part of the cohort received ICI therapy during the observation period as second-line therapy, either following a previous course of ICI therapy or chemotherapy, and a small number of patients received first-line ICI along with targeted therapy. While this imparts some level of treatment heterogeneity, the fact that the classifier still performed well is a testimony to its robustness. In addition, while sample sizes used for training, validation, and test were sufficiently large and representative of the full cohort with regard to the balance of demographic and clinical parameters, a larger sample size would be desirable for increased statistical power and potential fine-tuning of the algorithm. Lastly, this retrospective observational study, while showing clinical validity with two independent cohorts, necessitates prospective validation in a larger cohort to fully demonstrate clinical utility.

In conclusion, based on the data presented here, we propose that glycoproteomic profiling of blood provides a promising novel approach to guide clinical use of ICI therapy in patients with metastatic melanoma. Next steps might involve the development of glycoproteomic biomarkers in other tumor indications treated with ICI, or in adjuvant therapy. Analysis of longitudinal samples might also be helpful in segmenting patients that respond to ICI treatment and present with stable disease. The utility of glycoproteomic classifiers might also be explored for optimal treatment selection that accounts for tumor aggressiveness and potential adverse events including, for example, for the identification of patients that may benefit from ipilimumab/nivolumab combination, nivolumab/relatlimab combination, or nivolumab monotherapy.

## Data availability statement

The data presented in the study are deposited in the MassIVE repository, accession number MSV000092069.

## Ethics statement

The studies involving human participants were reviewed and approved by Massachusetts General Hospital Institutional Review Board protocols 11-488 and 11-181, and by Central Adelaide Local Health Network Human Research Ethics Committee (protocol HREC/16/RAH/95). The patients/participants provided their written informed consent to participate in this study.

## Author contributions

Conceptualization: AM, DS, KL, FS. Sample acquisition and clinical annotation: LE, MB, GT-R, DF, GB. Methodology: CP, PA, GX, AM, RR, XC, DS, KL, FS. Data generation and analysis: CP, PA, GX, AM, RR, DS, FS. Manuscript writing and reviewing: CP, PA, GX, KL, FS, DS. All authors contributed to the article and approved the submitted version.
