# Supplementary material for: Plasma glycoproteomic biomarkers identify metastatic melanoma patients with reduced clinical benefit from immune checkpoint inhibitor therapy
Source: Front Immunol. 2023 Jun 14;14:1187332. doi: 10.3389/fimmu.2023.1187332 (PMC10302726; doi:10.3389/fimmu.2023.1187332)

**Supplementary Material**

**Plasma glycoproteomic biomarkers identify metastatic melanoma patients with reduced clinical benefit from checkpoint inhibitor therapy**

Chad Pickering^1^, Paul Aiyetan^1^, Gege Xu^1^, Alan Mitchell^1^, Rachel Rice^1^, Yana G. Najjar^2^, Joseph Markowitz^3,4^, Lisa M. Ebert^5,6,7^, Michael P Brown^5,6,7^, Gonzalo Tapia-Rico^6,8^, Dennie Frederick^9^, Xin Cong^1^, Daniel Serie^1^, Klaus Lindpaintner^1^, Flavio Schwarz^1,^*, Genevieve M. Boland^9^

^1^InterVenn Biosciences, South San Francisco, CA, USA

^2^UPMC Hillman Cancer Center, Pittsburgh, PA USA

^3^Department of Cutaneous Oncology, H. Lee Moffitt Cancer Center and Research Institute, Tampa, FL, USA

^4^Immuno-Oncology Program, H. Lee Moffitt Cancer Center and Research Institute, Tampa, FL, USA

^5^Centre for Cancer Biology, SA Pathology and University of South Australia, Adelaide, SA, Australia

^6^Cancer Clinical Trials Unit, Royal Adelaide Hospital, Adelaide, SA, Australia

^7^Adelaide Medical School, The University of Adelaide, SA, Australia

^8^Present address: Icon Cancer Centre, Adelaide, SA, Australia

^9^Department of Surgery, Massachusetts General Hospital, Boston, MA, USA

*Corresponding author: flavio.schwarz@venn.bio

**Supplementary Tables**

**Supplementary Table 1.** Univariate Cox regression with respect to OS without and with adjustment for classifier prediction (likely/unlikely to benefit) in the discovery cohort. Variables that reached p<0.15 in both frameworks (marked with an asterisk) are included in multivariate modeling shown in Table 3. Global p-values for categorical variables take missing values into account.

|  | **Without adjustment** | | **With adjustment** | |
| --- | --- | --- | --- | --- |
| **Variable** | **HR (95% CI)** | **P-value** | **HR (95% CI)** | **P-value** |
| **Age (continuous years)*** | 1.018 (1.003, 1.033) | 0.017 | 1.015 (1.00, 1.03) | 0.057 |
| **Male sex (ref: female)** | 0.851 (0.574, 1.262) | 0.422 | 0.759 (0.509, 1.13) | 0.174 |
| **Pembrolizumab treatment**  **(ref: ipi/nivo)** | 0.89 (0.615, 1.289) | 0.539 | 0.935 (0.645, 1.355) | 0.722 |
| **Positive *BRAF* status**  **(ref: negative)*** | 0.689 (0.454, 1.045) | 0.08 | 0.718 (0.472, 1.09) | 0.119 |
| **LDH category*** |  | 7.3×10^-3^ |  | 0.101 |
| <ULN | Reference | | | |
| 1-2xULN | 1.417 (0.939, 2.138) | 0.097 | 1.382 (0.916, 2.086) | 0.123 |
| >2xULN | 2.711 (1.579, 4.656) | 3.0×10^-4^ | 2.007 (1.142, 3.53) | 0.016 |
| **ECOG performance status*** |  | 2.2×10^-4^ |  | 0.112 |
| 0 | Reference | | | |
| 1 | 1.353 (0.905, 2.022) | 0.14 | 1.307 (0.874, 1.955) | 0.193 |
| ≥2 | 4.352 (2.305, 8.216) | 5.8×10^-6^ | 2.372 (1.167, 4.823) | 0.017 |
| **M1 stage (ref: M0)*** | 2.43 (0.99, 5.963) | 0.053 | 2.284 (0.930, 5.609) | 0.072 |
| **Non-cutaneous subtype**  **(ref: cutaneous)*** | 2.149 (1.479, 3.121) | 5.9×10^-5^ | 2.099 (1.444, 3.052) | 1.0×10^-4^ |
| **Not first-line therapy**  **(ref: first-line)*** | 1.352 (0.906, 2.018) | 0.14 | 1.434 (0.958, 2.144) | 0.08 |

**Supplementary Table 2.** Demographic and clinical covariates in the discovery cohort stratified by classifier prediction. Counts are followed by the appropriate column-wise percentage, while continuous variables are summarized by medians and either IQRs or, for time to event variables, 95% confidence limits (NR = not reached).

| **Variable** | **Full cohort** | **Likely to benefit** | **Unlikely to benefit** |
| --- | --- | --- | --- |
| **Sample size** | 202 | 179 (89) | 23 (11) |
| **Male sex** | 139 (69) | 123 (69) | 16 (70) |
| **Age, yrs.** (continuous) | 65 (57, 73) | 65 (56.5, 73) | 68 (59.5, 78.5) |
| **Current ICI treatment** |  |  |  |
| Pembrolizumab monotherapy | 109 (54) | 98 (55) | 11 (48) |
| Nivolumab with/without ipilimumab combination | 93 (46) | 81 (45) | 12 (52) |
| **Survival-related events** |  |  |  |
| Progression (PFS) event | 145 (72) | 124 (69) | 23 (91) |
| Death (OS) event | 113 (56) | 92 (51) | 23 (91) |
| **Time to event, mos.** (continuous) |  |  |  |
| Progression | 5.5 (3.0, 9.9) | 7.6 (4.0, 16.9) | 2.1 (1.3, 3.0) |
| Death | 40.1 (27.3, 59.0) | 54.3 (37.9, NR) | 3.7 (2.4, 10.8) |
| ***BRAF* status** |  |  |  |
| Positive/mutant | 67 (33) | 63 (35) | 4 (17) |
| V600 | 57 (28) | 53 (30) | 4 (17) |
| Non-V600 | 9 (4) | 9 (5) | 0 (0) |
| Non-specific mutant | 1 (0) | 1 (1) | 0 (0) |
| Negative/wild type | 121 (60) | 104 (58) | 17 (74) |
| Missing | 14 (7) | 12 (7) | 2 (9) |
| **LDH, units/L** (continuous) | 206 (167, 281) | 200 (166, 266.8) | 357 (210, 792) |
| **LDH** (categorical) |  |  |  |
| <ULN | 105 (52) | 99 (55) | 6 (26) |
| 1-2xULN | 63 (31) | 57 (32) | 6 (26) |
| >2xULN | 27 (13) | 16 (9) | 11 (48) |
| Missing | 7 (3) | 7 (4) | 0 (0) |
| **M Stage** |  |  |  |
| M0 | 16 (8) | 15 (8) | 1 (4) |
| M1 | 186 (92) | 164 (92) | 22 (96) |
| M1a | 9 (4) | 8 (4) | 1 (4) |
| M1b | 31 (15) | 30 (17) | 1 (4) |
| M1c | 84 (42) | 73 (41) | 11 (48) |
| M1d | 62 (31) | 53 (30) | 9 (39) |
| **ECOG performance status** |  |  |  |
| 0 | 119 (59) | 113 (63) | 6 (26) |
| 1 | 67 (33) | 58 (32) | 9 (39) |
| ≥2 | 14 (7) | 8 (4) | 6 (26) |
| Missing | 2 (1) | 0 (0) | 2 (9) |
| **Melanoma subtype** |  |  |  |
| Cutaneous | 127 (63) | 116 (65) | 11 (48) |
| Mucosal | 20 (10) | 18 (10) | 2 (9) |
| Uveal | 15 (7) | 12 (7) | 3 (13) |
| Acral | 5 (2) | 5 (3) | 0 (0) |
| Unknown primary | 35 (17) | 28 (16) | 7 (30) |
| **Line of therapy** |  |  |  |
| First-line | 147 (73) | 131 (73) | 16 (70) |
| Second-line or later | 54 (27) | 48 (27) | 6 (26) |
| Missing | 1 (0) | 0 (0) | 1 (4) |

**Supplementary Table 3.** Demographic and clinical covariates in the external validation cohort stratified by classifier prediction. Counts are followed by the appropriate column-wise percentage, while continuous variables are summarized by medians and either IQRs or, for time to event variables, 95% confidence limits (NR = not reached).

| **Variable** | **Full cohort** | **Likely to benefit** | **Unlikely to benefit** |
| --- | --- | --- | --- |
| **Sample size** | 27 | 23 (85) | 4 (15) |
| **Male sex** | 19 (70) | 15 (65) | 4 (100) |
| **Age, yrs.** (continuous) | 71 (66, 81) | 70 (65.5, 81) | 74.5 (70.5, 79.5) |
| **Current ICI treatment** |  |  |  |
| Pembrolizumab monotherapy | 23 (85) | 19 (83) | 4 (100) |
| Ipilimumab/nivolumab combination | 4 (15) | 4 (17) | 0 (0) |
| **Survival-related events** |  |  |  |
| Progression (PFS) event | 10 (37) | 6 (26) | 4 (100) |
| Death (OS) event | 9 (33) | 6 (26) | 3 (75) |
| **Time to event, mos.** (continuous) |  |  |  |
| Progression | NR (9.9, NR) | NR (15.8, NR) | 2.6 (1.4, NR) |
| Death | 18.6 (15.8, NR) | NR (15.8, NR) | 6.0 (2.4, NR) |
| **Best overall response** |  |  |  |
| Complete response | 5 (19) | 5 (22) | 0 (0) |
| Partial response | 5 (19) | 5 (22) | 0 (0) |
| Stable disease | 6 (22) | 6 (26) | 0 (0) |
| Progressive disease | 9 (33) | 5 (22) | 4 (100) |
| Missing | 2 (7) | 2 (9) | 0 (0) |

**Supplementary Table 4**. At adjusted p<0.05, 21 functional pathways appear to be enriched; the top 20% of these are shown below.

| **ID** | **Description** | **Adjusted p** |
| --- | --- | --- |
| R-HSA-114608 | Platelet degranulation | 0.00e+00 |
| R-HSA-76005 | Response to elevated platelet cytosolic Ca2+ | 0.00e+00 |
| R-HSA-76002 | Platelet activation, signaling and aggregation | 0.00e+00 |
| R-HSA-8957275 | Post-translational protein phosphorylation | 3.52e-05 |
| R-HSA-6798695 | Neutrophil degranulation | 3.52e-05 |

**Supplementary Table 5**. At adjusted p<0.05, 21 functional pathways appear to be enriched; the top 20% of these and their associated genes are shown below. The Gene Ratio ‘proportion’ represents the ratio of the number of genes belonging to the pathway to the number of genes mapped to the reference database from the population of genes of interest (see ‘Features-related Genes’ Table).

| **ID** | **Gene ID** | **Gene Ratio** |
| --- | --- | --- |
| R-HSA-114608 | SERPINA1/SERPINA3/ORM1/APOA1/APOH/AHSG/ FN1/HRG/ITIH4/SELENOP/TF | 11/21 |
| R-HSA-76005 | SERPINA1/SERPINA3/ORM1/APOA1/APOH/AHSG/ FN1/HRG/ITIH4/SELENOP/TF | 11/21 |
| R-HSA-76002 | SERPINA1/SERPINA3/ORM1/APOA1/APOH/AHSG/ FN1/HRG/ITIH4/SELENOP/TF | 11/21 |
| R-HSA-8957275 | SERPINA1/APOA1/AHSG/FN1/TF | 5/21 |
| R-HSA-6798695 | SERPINA1/LRG1/SERPINA3/ORM1/B2M/AHSG/ HBB/TTR | 8/21 |

**Supplementary Figures**

**Supplementary Data Figure 1**. Kaplan-Meier curves of OS in the discovery cohort stratified by **a**) melanoma subtype, **b**) LDH category, **c**) ECOG performance status, and **d**) *BRAF* status.

**
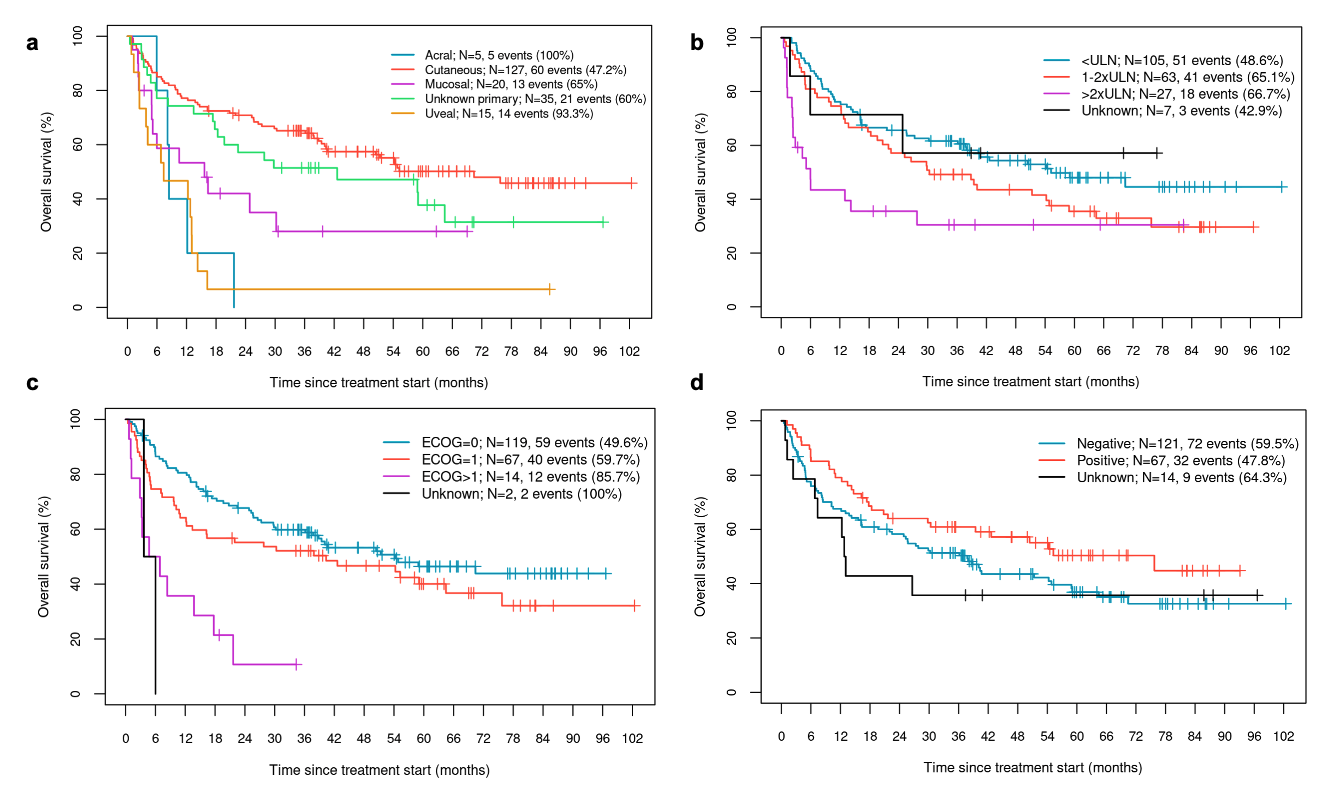
**

**Supplementary Figure 2**. Kaplan-Meier curves stratified by early failure (EF, death within six months of treatment start, n=40) and sustained controls (SC, progression- and death-free beyond three years of treatment; n=56) in the discovery cohort. “Other” defines intermediate phenotypes (n=106). **a**) PFS and **b**) OS respectively. **c**) Heatmap of 143 hierarchically-clustered concentration-normalized features that achieve FDR<0.05 in age- and sex-adjusted differential expression comparing early failures (EF) and sustained controls (SC).


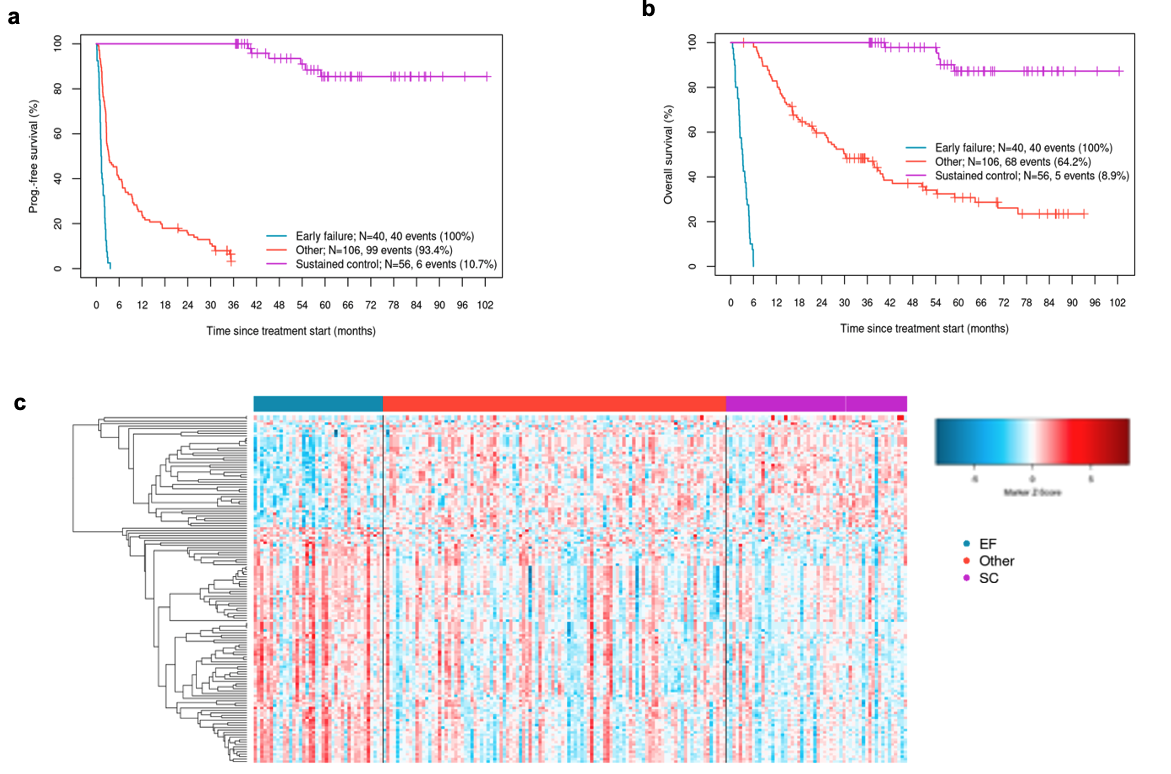


**Supplementary Figure 3**. Kaplan-Meier curves in the full discovery cohort stratified by classifier prediction and **a**) LDH category, **b**) ECOG performance status, and **c**) *BRAF* status.


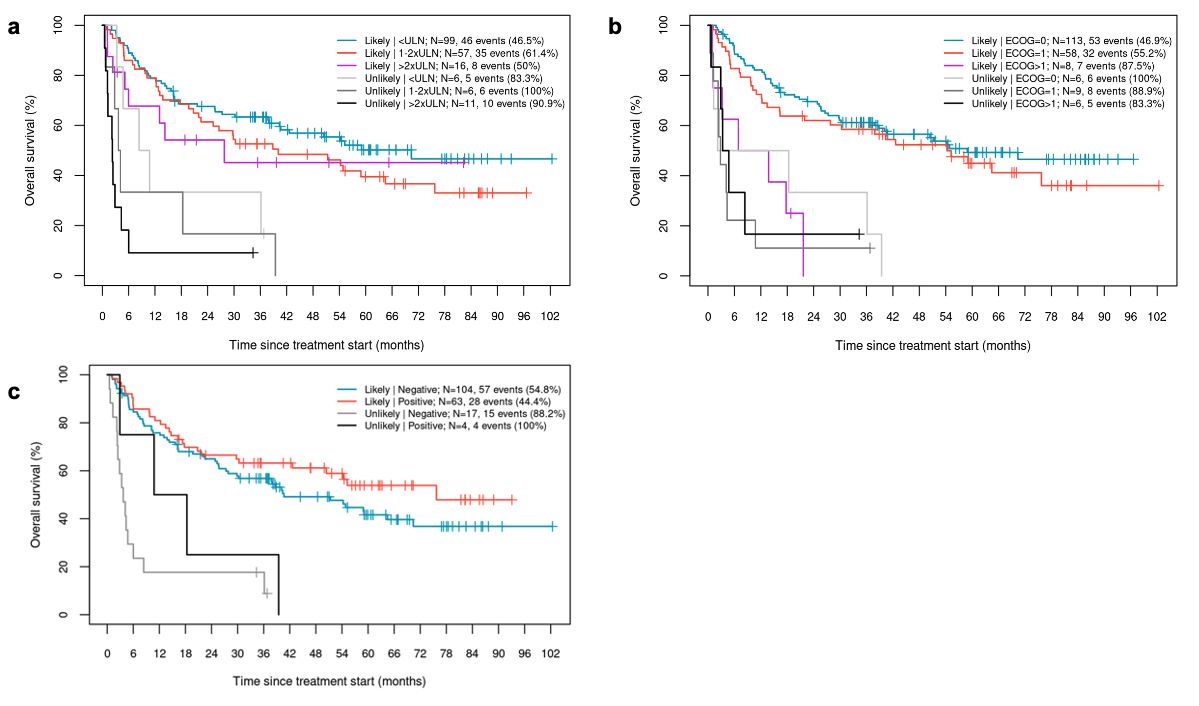

Supplement: Supplementary file 1 [file DataSheet_1.docx]
